# Long-Lasting Insecticide Net Ownership, Access and Use in Southwest Ethiopia: A Community-Based Cross-Sectional Study

**DOI:** 10.3390/ijerph14111312

**Published:** 2017-10-27

**Authors:** Dinberu Seyoum, Niko Speybroeck, Luc Duchateau, Patrick Brandt, Angel Rosas-Aguirre

**Affiliations:** 1 Institute of Health and Society (IRSS), Université Catholique de Louvain, 1200 Brussels, Belgium; speybroeck@uclouvain.be; 2 Department of Statistics, Natural Science College, Jimma University, Jimma 378, Ethiopia; 3 Biometrics Research Group, Faculty of Veterinary Medicine, Ghent University, 9000 Ghent, Belgium; Luc.Duchateau@ugent.be; 4 School of Economic, Political and Policy Sciences, The University of Texas, Dallas, TX 75080, USA; pbrandt@utdallas.edu; 5 Institute of Tropical Medicine “Alexander von Humboldt”, Universidad Peruana Cayetano Heredia, Lima 31, Peru; angelrosasa@gmail.com

**Keywords:** long lasting insecticide treated net, access, ownership, LLIN use, Ethiopia

## Abstract

***Introduction*:** A large proportion of the Ethiopian population (approximately 68%) lives in malaria risk areas. Millions of long-lasting insecticide treated nets (LLINs) have been distributed as part of the malaria prevention and control strategy in the country. This study assessed the ownership, access and use of LLNs in the malaria endemic southwest Ethiopia. ***Methods:*** A community-based cross-sectional study was conducted in southwest Ethiopia during October–November 2015, including 836 households from sixteen villages around Gilgel-Gibe dam area. Indicators of ownership, access and use of LLINs were derived following the Roll Back Malaria (RBM) guidelines. Factors associated with failure for both LLIN access and use were analysed at household level using a multivariate logistic regression model. ***Results:*** The proportion of households with at least one LLIN was 82.7% (95% CI: 80.0, 85.1). However, only 68.9% (95% CI: 65.6, 71.9) had enough LLINs to cover all family members (with ≥one LLIN for every two persons). While 75.3% (95% CI: 68.4, 83.0) of the population was estimated to have accessed to LLINs, only 63.8% (95% CI: 62.3, 65.2) reported to have used a LLIN the previous night. The intra-household gap (i.e., households owning at least one LLIN, but unable to cover all family members) and the behavioral gap (i.e., household members who did not sleep under a LLIN despite having access to one) were 16.8% and 10.5%, respectively. Age, marital status and education of household heads, as well as household size and cooking using firewood were associated with the access to enough LLINs within households. Decreased access to LLINs at households was the main determinant for not achieving ≥80% household members sleeping under a LLIN the previous night. Other associated factors were household size and education level of household head. ***Conclusions:*** LLIN coverage levels in study villages remain below national targets of 100% for ownership and 80% for use. The access to enough LLINs within the households is the main restriction of LLIN use in the study area.

## 1. Introduction

Malaria is one of the most important public health problems worldwide with about 3.5 billion people living at malaria risk in 2015, and millions of them still not accessing preventive and control measures, especially in sub-Saharan African countries [1]. In Ethiopia, about 68% of the total population resides in areas with high malaria risk [2], and 2,174,707 cases and 662 deaths due to malaria were reported in 2014–2015 [3]. 

Insecticide-treated bed nets (ITNs), more specifically long lasting insecticide-treated nets (LLINs), are known to be highly effective in reducing malaria morbidity and mortality [4,5,6]. The World Health Organization (WHO) recommends achieving high LLIN coverage rates in endemic areas through free-of-charge or highly subsidized delivery of LLINs to maximize the effectiveness of control programs [7]. According to WHO estimates [1], control interventions averted 663 million malaria cases in sub-Saharan Africa between 2001 and 2015 [1], and the use of LLINs contributed about 69% to those averted cases. However, reaching universal coverage and use of LLINs remains challenging in the African continent. In 2015, 67% of households had access to an ITN but only 55% of the population slept under an ITN the previous night [1]. 

The distribution of LLINs is a key intervention for preventing malaria disease in Ethiopia [7,8,9,10]. According to the Ethiopian Ministry of Health (MoH), near to 20 million LLINs were delivered in the country between 2013 and 2015 with participation of health workers, community volunteers, and the local authorities [2,7]. Despite these efforts, Ethiopian malaria goals for 2015 in malaria endemic areas (i.e., 100% of LLIN coverage, and more than 80% of use of LLINs) have not been achieved [11,12,13,14]. The percentage of households with at least one LLIN at country level was 64.0% in 2015, and regions such as Oromia presented even lower LLIN coverage than the national average [8]. 

In addition to periodic data from household surveys aiming to assess whether the population at risk receives enough nets and uses them properly [15], the design of effective strategies based on the distribution of ITNs/LLINs requires information about the factors that can affect the success and failure for getting high levels of LLIN access, ownership and use [1,4,16]. LLIN access is measured at household level and takes into account the number of available LLINs for the total household members, but very few studies have investigated risk factors associated with poor access to LLINs (i.e., less than one LLIN for every two household members) [5,14,17,18]. Instead, several studies have explored the determinants of ITN/LLIN ownership and use. The demographics, the socio-economic status, the knowledge about malaria and the protective effect of LLINs, and the intensity of malaria transmission had been identified as factors for owning LLIN in eastern and southwest Ethiopia [19,20]. Besides the demographic characteristics of individuals, the sleeping patterns, the family size, and the net type have been also reported among the factors associated with the LLIN use in Amhara Regional States of Ethiopia and western Kenya [9,14,21,22]. Here, we present a cross-sectional study that reports the levels of ownership, access and use of LLINs in southwest Ethiopia in 2015. We identify the factors that predict failure of LLIN access and use.

## 2. Materials and Methods 

### 2.1. Study Area

**Figure 1:**
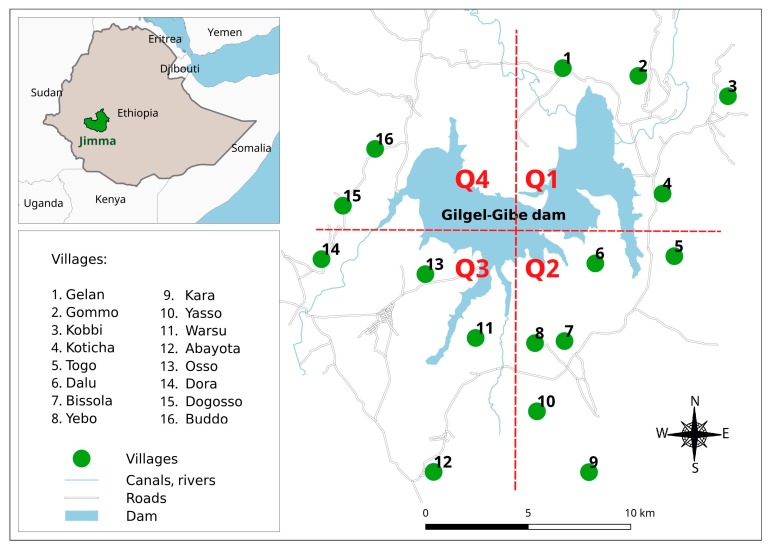
Map showing Jimma zone and study villages around the Gilgel-Gibe hydroelectric dam reservoir, Jimma zone, Ethiopia. Q1 (Quadrant 1) includes Gelan, Gommo, Kobbi and Koticha villages; Q2 (Quadrant 2) includes Togo, Dalu, Bissola, Yebo, Kara and Yasso villages; Q3 (Quadrant 3) includes Dora, Osso, Warsu and Abayota villages; Q4 (Quadrant 4) includes Buddo, and Dogooso villages.

The study was conducted in Jimma zone, Oromia region of Ethiopia, 260 km southwest of Addis Ababa, at an altitude of 1734–1864 m above sea level, between latitudes 7°42′50″ N and 07°53′50″ N and between longitudes 37°11′22″ E and 37°20′36″ E. Among all villages located within a 10 km radius (265–9046 m) from the Gilgel-Gibe dam, sixteen villages were randomly selected for a series of studies aimed to assess the impact of the dam on the health and other sectors (environment, agriculture and economy) following its starting operation in 2004 [8,23,24,25]. The distribution of these villages by quadrant in the study area is: Quadrant 1 (Q1) in the northeast including Gelan, Gommo, Kobbi and Koticha villages; Quadrant 2 (Q2) in the southeast including Togo, Dalu, Bissola, Yebo, Kara and Yasso; Quadrant 3 (Q3) in the southwest including Dora, Osso, Warsu and Abayota; and Quadrant 4 (Q4) in the northwest including Buddo, and Dogooso villages (Figure 1). Malaria transmission in the area is unstable and seasonal like in other parts of the country [22,23,24,25], with *P. falciparum* and *P. vivax* accounting respectively for 64% and 36% of malaria infections [23]. A previous cohort study that followed up children <10 years for two years (2008–2010) in the same study area found spatial clusters of higher malaria incidence in Q2 and Q4 [25]. Main economic activities include the cultivation of staple crops (maize, teff and sorghum), cattle and small stock-raising. Most Residents in the study villages belong to the Oromo ethnic group, which is one of the largest ethnic groups in Ethiopia.

### 2.2. Study Design and Population

A cross-sectional survey was conducted in October–November 2015 (high transmission season) in the study area. The required sample size for the study was 833 households, assuming that 56% of households owned at least one LLIN [26] and using a precision of 5%, a significance level of 5%, a power of 80%, and a non-response rate of 10% [27]. This sample was equally distributed among the villages, and households in each village were randomly selected from the complete list of households provided by the village administrative offices. A household was defined as any unit headed by a male or female with his/her dependents and/or spouse, and who share a cooking pot/common eating place and sleep under the same roof.

### 2.3. Data Collection

Trained field workers with secondary school level or higher (under supervision of a field coordinator) were in charge of the data collection, using a structured questionnaire and an observational checklist. The English version of the questionnaire was translated into the local language (“Amharic” and “Afan Oromo”) and then validated through a pilot-evaluation in a malaria endemic area near the study area. Permission from the village zonal and district administrative offices was obtained before the onset of the study. Each selected household was visited, and a written informed consent was sought from the head of the household. Data were collected on demographics, socio-economic status, housing structure and construction materials, cooking and live stocking practices, history of malaria in the past year, discussion/conversation about malaria at household in the past month, as well as on ITN/LLIN ownership, access and use. ITN/LLIN net use the night before the survey was initially established by respondent self-report, and then confirmed after visually verifying that the LLINs were hanging above sleeping spaces. 

### 2.4. Indicators of LLIN Coverage, Access and Use

Seven indicators were calculated in the study:

*Proportion of households with at least one LLIN (I_1_).* This indicator provides a measure for household ownership of an LLIN. The numerator includes all households with at least one LLIN and the denominator is the total of number of sampled households [23].

*Proportion of households with at least one LLIN for every two family members (I_2_)*. This indicator measures the proportion of households that have enough access to LLIN, i.e., those households with a sufficient number of LLINs to protect all household members. For its calculation, the number of LLIN owned by the household is divided by number of household members. Then, the numerator includes all households that have a LLIN to people ratio of 0.5 or higher, and the denominator is simply the total number of households surveyed [3,28]. 

*Proportion of individuals with access to LLIN within the households (I_3_)*. This indicator estimates the proportion of population that could potentially be covered by the existing LLINs, assuming that each LLIN in a household can be used by two people. The numerator includes all household members who had access to a LLIN, and the denominator is the de-facto population in the sample. The calculation of the indicator was done in two steps [1,7]. First, an intermediate variable “potential LLIN users” was calculated by multiplying the number of LLINs in each household by two. To correct for households with more than one net for every two people, the potential LLIN users were set equal to the members in that household if the potential users exceeded the number of people in the household. Second, the indicator for individual access was calculated by dividing the potential LLIN users by the number of individuals in each household. 

*Proportion of households with at least one LLIN for every two people among households owning any LLIN (I_4_)*. This indicator measures the saturation with LLIN in households with any LLIN. Then, the reverse of this indicator (1-*I_4_*) represents the intra-household LLIN ownership gap, i.e., households owning at least one LLIN, but that are not able to cover all household members [29]. 

*Proportion of individuals who slept under LLINs the previous night (I_5_)*. This indicator measures the level of LLIN use in all individuals at the time of the survey. The numerator contains all individuals who slept under a LLIN prior the survey, and the denominator includes the total surveyed population. 

*Proportion of individuals sleeping under an ITN the previous night among those with access* (*I_6_*). This indicator is obtained by dividing the number of people who slept under LLIN the previous night among those with access by the total population who had access to an LLIN. As shown in the description of *I*_3_, populations with access are the potential LLIN users taking into account that one LLIN is for every two people. Then, the reverse of this indicator (1-*I*_6_) is known as the LLIN behavioral gap, i.e., the proportion of household members who did not sleep under an ITN despite having access to one.

*Ratio of LLIN use to LLIN access (I_7_).* This alternative indicator compares the indicator of individual LLIN use against the indicator of individual LLIN access (*I*_7_ = *I*_5_/*I*_3_*)*, and quickly identifies if the differences between those indicators are mainly explained by behavioral factors.

### 2.5. Data Analysis

The data were entered and cleaned in Excel spread sheets (Microsoft Corp, Redmond, WA, USA), and the analysis was performed with STATA v.14.0 (Stata Statistical Software: Release 14, College Station, TX, USA: StataCorp LP), using the command “svy” [30]. LLIN indicators and corresponding two-sided 95% confidence intervals (CI) were estimated overall and by quadrant, taking into account the survey design characteristics: village as strata, household as primary sampling unit, and corresponding sampling weights (ratio between the sampled households and the total number of households in the village). Univariate and multivariate analysis were performed using survey logistic regression to assess risk factors for households that did not reach at least one LLIN for every two family members (i.e., failure for household access to LLIN), as well as, for households in which the proportion of members that slept under a LLIN the previous night did not reach 80% (i.e., failure for using LLIN at household). The following potential risk factors were evaluated: demographic characteristics of household head (i.e., age, sex, education level and marital status), housing structure (i.e., predominant material in walls and roof), family size and practices (i.e., cooking practices and livestock ownership), history of malaria in household head and family members in the past 12 months, and discussion/conversation about malaria at household in the past month. Factors with *p* < 0.2 for the likelihood ratio test in the univariate survey analysis (intended to assess enough potential risk factors) were considered for inclusion in the multivariate adjusted survey model. Using manual backward methods, final models retained all factors that were significantly associated with failure for household access to LLIN, and failure for using LLIN at household (*p* < 0.05). In addition, a linear regression analysis assessed how *I*_5_ (LLIN use) and *I*_7_ (ratio of LLIN use to LLIN access) may be affected by different values of *I*_3_ (LLIN access). 

## 3. Results

The study included 816 households (51 households in each of the 16 selected villages), accounting for 4323 individuals living in them (about 5.3 per household). With a mean age of 43 ± 13 years, most heads of households were male (86.9%) and had farming (96.7%) as their main economic activity. Houses were mainly built of mud walls (98.5%) and thatched roofs (65.2%). The main fuel used for cooking (53.2%) was firewood, and almost all households had livestock (94.9%). A total of 18.2% household heads and 14.6% family members reported to have experienced a malaria episode in the past 12 months. Demographic and household characteristics, as well as history of malaria in household members overall and by quadrant are presented in Supplementary Table S1. 

### 3.1. LLIN Ownership, Access and Use

In total 1982 ITNs were found in the study villages, from which 1974 (99.5%) were LLINs (about 1.9 LLINs per household). Table 1 and Table S2 show the estimated indicators of ownership, access and use by quadrant and village, respectively. Overall household ownership of LLINs (*I*_1_) reached 82.7% (95% CI: 80.2, 85.1), ranging from 72.2% (95% CI: 62.4, 80.2) in villages of Quadrant 4 to 88.0% (95% CI: 83.9, 91.2) in villages of Quadrant 2. The overall proportion of households with enough LLINs (one LLIN for every two household member) for all their members (*I*_2_) was 68.9% (95% CI: 65.6, 71.9), with the lowest values in Quadrant 4 (61.5%) and the highest ones in Quadrant 2 (71.2%). 

The overall proportion of population with LLIN access (*I*_3_) was 75.3% (95% CI: 68.4, 83.0), while the proportion of population who actually used an LLIN the previous night (*I*_5_) was 63.8% (95% CI: 62.3, 65.2). For both indicators (i.e., *I*_3_ and *I*_5_), the highest values were found in Quadrant 2 and the lowest ones in Quadrant 4. The proportion of households with at least one LLIN for every two people among households owning LLIN (*I*_4_) was above 80% in all quadrants (overall average: 83.2; 95% CI: 80.3, 85.9), therefore the overall intra-household LLIN ownership gap (1-*I*_4_) was calculated as 16.8%. On the other hand, the proportion of population sleeping under a LLIN the previous night among those with access was calculated in 89.5%, thus the LLIN behavioral gap (1-*I*_6_) was 10.5%. Moreover, the overall ratio of LLIN use to LLIN access (*I*_7_ = *I*_5_*/I*_3_) was estimated as 0.85.

**Figure 2:**
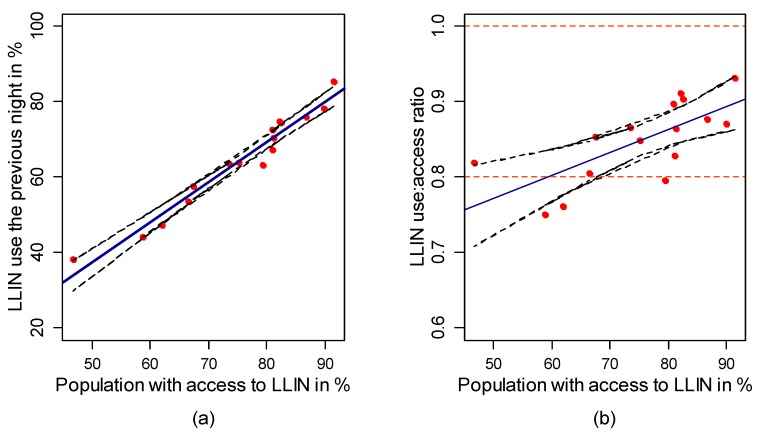
(**a**) Relationship between population LLIN use and population access; and (**b**) relationship between the ratio of population LLIN use to access, and population LLIN access. The solid blue line represents the predicted values by the linear regression and the black dashed lines are the 95% confidence intervals of those values. Red points are observed values.

A regression model that explains LLIN use (*I*_5_) as function of LLIN access (*I*_3_) predicted that about 95.1% of the population would use LLINs if the access were universal (100% of access) (Figure 2a, *p* < 0.001). On the other hand, the regression model that describes the ratio of LLIN use to LLIN access (*I*_7_) as function of LLIN access (*I*_3_) (Figure 2b) indicated that population access to LLINs (*I*_3_) between 60% and 100% would allow achieving ratios of LLIN use to access between 0.8 and 0.9. 

### 3.2. Risk Factors for Failure of Household Access to LLINs and Using LLINs at Household Level 

Age, education, and marital status of household heads, as well as family size and cooking using firewood remained independent risk factors for the failure of a household access to LLINs in the final multivariate model. Households in which heads were between 41 and 50 years old (Adjusted Odds Ratio (AOR) = 1.7; 95% CI: 1.1, 2.8) and older than 50 years old (AOR = 1.9; 95% CI: 1.2, 3.1) were more likely to fail to access LLINs compared to those in which heads were younger than 36 years. Failure of LLIN access was also more common in households where heads were single or divorced (AOR = 3.8, 95% CI: 1.6, 9.0), and had only primary education level (AOR = 2.6; 95% CI: 1.2, 5.6), or no education (AOR = 3.1; 95% CI: 1.6, 6.2) compared to households where heads were married and had higher education level, respectively. Households with large families (≥7 members) had higher odds of failure to access LLIN (AOR = 9.3; 95% CI: 5.2, 16.8) than households with less than 4 members. In addition, households that used firewood as fuel for cooking were more likely to fail to access LLINs (AOR = 1.7; 95% CI: 1.2, 2.4) compared to those that use other types of fuel (Table 2). 

The main independent determinant of failure for using LLINs in a household (i.e., <80% of household members that slept under a LLIN the previous night) was the household’s access to LLINs. Households with less than one LLIN for every two family members had higher odds (AOR = 36.5; 95% CI: 18.5, 71.8) to fail in using LLIN than households with one or more LLIN for every two members. Other factors that remained significant in the final multivariate model for failure for LLIN use at household were the family size and the education level of household head. Indeed, households with 4–6 members (AOR = 7.4, 95% CI: 2.6, 21.3) and those in which heads had no education (AOR = 2.3, 95% CI: 1.2, 4.8) had, respectively, higher odds of failure to have LLIN use than households with three or fewer members, and those in which heads had secondary or higher education level (Table 3).

## 4. Discussion

This paper reports the current indicators for LLIN ownership, access and use in a malaria endemic-prone area of southwest Ethiopia, calculated following the recommendations of the Roll Back Malaria Monitoring and Evaluation Reference Group (MERG) [28]. The study area has reached high levels of LLIN ownership, even higher than the previously published regional (58.5%) and national (64%) estimates in 2015 [8]. The national distribution of LLINs in the past three years (about 20 million LLIN between 2014 and 2015) may have contributed to this achievement [8]; however, LLIN ownership levels remained still far below the national goal for 2015 of getting universal coverage (100%) of LLINs in malaria endemic areas (i.e., all sleeping spaces with LLIN) [11]. Moreover, although the proportion of people (63.8%) who used LLIN the previous night in our study was higher than the recent reported figures (33.5%) of a study in eastern Ethiopia [31], this indicator of LLIN use still remains below the optimal threshold suggested by WHO (80%) [2,32].

For many years, the outcomes of malaria control with ITNs/LLINs have been assessed using mainly two indicators: the proportion of households with at least one LLIN (household LLIN ownership, *I*_1_) and the proportion of individuals who slept under LLINs the previous night (individual LLIN usage, *I*_4_) [28,33]. However, several studies in endemic areas have described low levels of individual LLIN use (*I*_5_) despite the presence of fair levels of household LLIN ownership (*I*_1_) [12,14]. Some of these studies have remarked that the difference between both indicators was mainly explained by an insufficient availability of LLINs to cover all family members (mostly in large families) instead of the complete absence of LLIN at households [34,35]. Similarly, the indicator of individual LLIN use (*I*_5_ = 63.8%) in our study area was not as high as the indicator of household LLIN ownership (*I*_1_ = 82.7%), and indeed the estimated intra-household LLIN ownership gap (1*-I*_4_ = 16.8%) confirmed the need to orient the strategy of LLIN distribution with the aim of reaching enough LLINs to protect all family members at households instead of insuring at least one LLIN per household. 

Accordingly, WHO has recently recommended that delivery strategies of LLINs should ensure the availability of one LLIN for every two people [32], and the MERG has incorporated indicators that allow monitoring the access to LLIN appropriately at household (i.e., proportion of households with at least one LLIN for every two household members, *I*_2_) and at individual levels (proportion of individuals with access to LLIN within the household, *I*_3_). Therefore, the levels of both indicators in the study villages (68.9% and 75.3%, respectively) indicate that current strategies to deliver LLINs (mainly based on free mass distribution campaigns with the operational delivery criteria of one LLIN for every two people [36]) may not be enough to reach universal coverage of LLINs in malaria endemic areas of southwest Ethiopia. 

The proportion of the population sleeping under a LLIN the previous night among those with access (*I*_6_), the behavioral LLIN gap (1-*I*_6_) and the population LLIN use to access ratio (*I*_7_) provide complementary information regarding the population use of LLINs. The relative large value of the first indicator (~90%) and corresponding low behavioral gap (~10%) which contrast with the proportion of the total population sleeping under a LLIN the previous night (*I*_5_ = 62.9–68.4%) in most villages (Quadrants 1–3) further supports the argument that the limited use of LLINs by the population is mainly due to insufficient availability of LLINs in those villages (*I*_2_ = 68.6–71.2%) than due to poor population awareness concerning malaria prevention methods. Similarly, the population LLIN use to access ratio in those villages of around 0.85 confirms that there is not much room for improvement in the population use of LLINs, if only strategies aimed to improve the net use behavior are considered [37]. Conversely, a reduced access to LLIN (*I*_2_ = 61.5%), limited community prevention practices evidenced through an appreciable behavioral LLIN gap (1-*I*_6_ = 16.4%) and a suboptimal LLIN use to access ratio (*I*_7_ = 0.78) may explain why the lowest values of LLIN use were found in villages of Quadrant 4. Noteworthy, the LLIN use to access ratio in Quadrant 4 was below the mean obtained (0.81) from the analysis of 41 Demographic Health Surveys (DHS) and Malaria Indicator Surveys (MIS) in sub-Saharan Africa [37]. 

Large household size (≥7 family members) as the main associated factor for households with insufficient access to LLINs (less than one LLINs for every two persons) is not surprising, and is in line with recent findings in Sierra Leone [38] and Burkina Faso [39] where available ITNs at household were not able to protect all family members. Besides household size, socio-demographic characteristics of household heads and cooking practices using firewood were also independently associated with limited LLIN access. As previously reported in southwest Ethiopia [26], the marital status of household heads was associated with the access to LLIN in our study, with households headed by single or divorced heads four times more likely to lack sufficient access to LLINs than households headed by married couples. A decreased decision-making power in households with a single-parent family structure may explain their lower LLIN access in comparison with household with two-parent families, which may lead to a lower retention of nets (after being delivered) and/or acquisition of nets (in the market) to protect family members against malaria [40]. Moreover, the relationship between the education level of household heads and LLIN ownership has also been studied across several countries of sub-Saharan Africa, with no conclusive results. While a study in eastern Ethiopia found that net ownership was not associated with the education level of household heads [41], other studies in Kenya and Tanzania have reported a significant correlation between education level of household heads and the possession of nets [19,42,43] similar to our study. A higher education level in household heads (as proxy of socioeconomic status) may reflect an increased economic power to acquire LLINs in the market [44], but also may contribute to a better family awareness concerning malaria prevention measures [45]. On the other hand, the lower access to enough LLINs in households with older heads has also been reported in southwest Ethiopia [26,44] and explained by the higher probability for older heads of having larger families. Interestingly, the reduced access to enough LLINs at households in which firewood is used as the main cooking fuel may be explained by a lower perception of the malaria risk due to a decrease in the human-vector contact rate within the house. The repelling effect of the smoke may reduce the indoor density of the malaria vector, giving a perception of protection among family members that likely affects the family decision about acquiring LLINs and/or keeping existing LLINs [46]. 

The Ethiopian government has committed to achieve malaria elimination in specific geographical areas with historically low malaria transmission and near zero malaria deaths in remaining areas with higher malaria transmission by 2015 [11]. To this effect, the country’s goal was that at least 80% of people at malaria risk use LLINs. In agreement with the analysis of LLIN indicators, the main independent determinant for reduced use of LLINs at households (<80% of household members using LLIN the previous night) in our study area was the insufficient access to LLINs. In other words, without appropriate and universal coverage of LLINs, the Ethiopian malaria control goals for LLIN use will not be reached. Besides strengthening the periodical free mass distribution campaigns of LLINs, there is the need to improve the contribution of the routine distribution through the antenatal care and immunization campaigns [47], as well as to develop alternative strategies such as the distribution of LLINs in schools and the opportune replacement of LLINs by community health workers [48]. 

The significant reduced use of LLINs in households where heads had no education may be explained by the limited knowledge and wrong perception of family members about malaria and its control, which have repeatedly been reported to be associated with low levels of ITN ownership and use [14,42,49,50]. It is well known that despite increasing coverage and ownership of LLINs, the consistent and correct use of the LLINs is not ensured [26]. Information, education and communication (IEC) and behavior change communication (BCC) interventions should always go along with mass distribution campaigns to encourage the correct hanging, preservation, washing, and use of available LLINs [51,52]. After the campaigns, appropriate community communication channels should be used to reinforce the importance of the LLINs for the malaria prevention, and to convince people to use them permanently [44,52]. Further research studies including qualitative methods are needed in Ethiopia to better understand how community perceptions and believes influence the sustained use of LLINs at household. 

## 5. Conclusions

Despite the progress made in the last decade, LLIN coverage levels in selected malaria endemic villages of southwest Ethiopia remain below national targets of 100% for ownership and 80% for use. The access to enough LLINs within the households as the main restriction of LLIN use in the study area highlights the need to strengthen the current mass distribution campaigns (ensuring always at least one LLIN for every two persons), but also to use alternative mechanisms for continuous distribution of LLINs (e.g., routine distribution through antenatal care, immunization, and replacement of LLINs by community voluntaries). Effective BBC interventions should be implemented before, during and after distribution campaigns to ensure the appropriate and permanent use of LLINs. 

## Figures and Tables

**Table 1:** Indicators of ownership, access and use of LLINs in the study area, southwest Ethiopia.

Indicator (Level)	Quadrant 1			Quadrant 2			Quadrant 3			Quadrant 4			Total		
	n/N	%	95% CI	n/N	%	95% CI	n/N	%	95% CI	n/N	%	95% CI	n/N	%	95% CI
*I*_1_ (HH)	166/204	80.8	(75.1, 85.6)	269/306	88.0	(83.9, 91.2)	168/204	81.1	(75.1, 85.9)	74/102	72.2	(62.4, 80.2)	677/816	82.7	(80.0, 85.1)
*I*_2_ (HH)	141/204	68.6	(62.1, 74.5)	217/306	71.2	(66.0, 76.1)	143/204	68.8	(62.1, 74.9)	62/102	61.5	(51.5, 70.6)	563/816	68.9	(65.6, 71.9)
*I*_3_ (Ind)	151/204	74.2	(68.5, 79.8)	243/306	79.5	(75.4, 83.4)	154/204	75.8	(70.2, 81.3)	65/102	64.2	(55.2, 73.2)	614.5/816	75.3	(68.4, 83.0)
*I*_4_ (HH)	141/166	84.8	(78.3, 89.6)	217/269	81.0	(75.9, 85.3)	143/168	84.8	(78.4, 89.6)	62/74	85.2	75.6, 91.5)	563/677	83.2	(80.3, 85.9)
*I*_5_ (Ind)	673/1070	62.9	(59.9, 65.8)	1104/1614	68.4	(66.1, 70.7)	689/1058	65.1	(62.2, 67.9)	292/581	50.2	(46.1, 54.3)	2758/4323	63.8	(62.3, 65.2)
*I*_6_ (Ind)	602/665	90.5	(87.9, 92.6)	918/1020	90.0	(87.9, 91.7)	583/641	90.9	(88.4, 93.0)	271/324	83.6	(79.1, 87.4)	2374/2650	89.5	(88.3, 90.7)
*I*_7_ ratio		0.85	(0.83, 0.88)		0.86	(0.85, 0.88)		0.86	(0.84, 0.89)		0.78	(0.74, 0.84)		0.85	(0.79, 0.91)

I: Indicators; HH: household; Ind: individual; n: number of HH or Ind that meet the indicator; N: total number of HH or Ind that were assessed.

**Table 2:** Univariate and multivariate risk factor analysis for failure to household access to ITNs in southwest Ethiopia (N = 816).

Covariates		Failure to HH Access to LLINs				Univariate			Multivariate	
		n	N	%	95% CI	OR	95% CI	*p*	AOR	95% CI
Location of the village	Quadrant 1	63	204	31.4	(25.5, 37.9)	1		0.35		
	Quadrant 2	89	306	28.7	(23.9, 33.9)	0.9	(0.6, 1.3)			
	Quadrant 3	61	204	31.2	(25.2, 38.0)	0.9	(0.6, 1.5)			
	Quadrant 4	40	102	38.5	(29.4, 48.5)	1.4	(0.8, 2.3)			
Gender of HH head	Female	30	103	29.6	(21.4, 39.4)	1		0.74		
	Male	223	713	31.3	(28.0, 34.8)	1.1	(0.7, 1.7)			
Age of the HH head	≤35	53	268	19.8	(15.5, 25.0)	1		<0.01	1	
	36–40	47	147	31.9	(24.8, 39.9)	1.9	(1.2, 3.0) *		1.0	(0.6, 1.6)
	41–50	80	205	39.9	(33.3, 46.9)	2.7	(1.8, 4.1) **		1.7	(1.1, 2.8) *
	>51	73	196	36.5	(29.9, 43.6)	2.3	(1.5, 3.6) **		1.9	(1.2, 3.1) *
Education of HH	Above primary	13	99	13.8	(8.1, 22.5)	1		<0.01	1	
	Primary	40	142	27.7	(20.9, 35.6)	2.4	(1.2, 6.3) *		2.6	(1.2, 5.6) *
	No education	200	575	13.8	31.1, 38.9)	3.4	(1.8, 6.3) **		3.1	(1.6, 6.2) *
Occupation of HH head	Farmer	245	789	31.1	(27.9, 34.4)	1		0.294		
	Government employee	3	17	19.3	(6.3, 46.0)	2.2	(0.6, 7.7)			
	Other	5	10	49.5	(21.8, 77.4)	4.1	(0.7, 24.1)			
Marital status of HH head	Married	224	729	30.6	(27.4, 34.0)	1		0.049	1	
	Widowed	14	54	25.9	(15.9, 39.5)	0.8	(0.4, 1.5)		1.3	(0.6, 2.8)
	Single/divorced	15	33	50.5	(33.8, 67.2)	2.3	(1.1, 4.7) *		3.8	(1.6, 9.0) *
History of malaria in the past year of HH head	No	192	664	29.2	(25.8, 32.8)	1		0.013	1	
	Yes	61	152	39.8	(32.2, 47.8)	1.6	(1.10, 2.33) *		1.1	(0.7, 2.0)
Family size	1–3 persons	24	138	18.1	(12.5, 25.7)	1		<0.01	1	
	4–6 persons	92	451	20.4	(16.9, 24.5)	1.15	(0.70, 1.91)		1.5	(0.9, 2.5)
	≥7 persons	137	227	60.5	(53.9, 66.7)	6.9	(4.1, 11.6) **		9.3	(5.2, 16.9) **
Predominant material in HH walls	Mud	248	805	31	(27.9, 34.3)	1		0.571		
	Cements	5	11	39	(15.9, 68.5)	1.4	(0.4, 4.9)			
Predominant material in HH roof	Iron	92	281	32.7	(27.3, 38.5)	1		0.496		
	Thatched	161	535	30.3	(26.5, 34.3)	0.8	(0.6, 1.2)			
Firewood use for cooking	No	118	436	27.4	(23.3, 31.8)	1		0.016	1	
	Yes	135	380	35.4	(30.6, 40.3)	1.5	(1.1, 2.0) *		1.7	(1.2, 2.4) *
Livestock ownership	No	10	40	28.3	(16.1, 44.8)	1		0.707		
	Yes	243	776	31.3	(28.1, 34.6)	1.2	(0.5, 2.4)			
History of malaria in the past year of any household member	No	199	693	28.8	(25.6, 32.3)	1		<0.01	1	
	Yes	54	123	44.4	(35.7, 53.5)	2.0	(1.3, 3.0) *		1.4	(0.9, 2.4)
Discussion about malaria in the past month	No	70	183	38.1	(31.3, 45.3)	1		0.021	1	
	Yes	183	633	29	(25.5, 32.7)	0.7	(0.5, 0.9) *		0.7	(0.5, 1.1)

HH: Household; * *p* < 0.05; ** *p* < 0.001.

**Table 3:** Univariate and multivariate analysis risk factor analysis for failure for using ITN at household in southwest Ethiopia (N = 816).

Covariates		Failure for Using LLINs				Univariate			Multivariate	
		n	N	%	95% CI	OR	95% CI	*p*	AOR	95% CI
Location of the village	Quadrant 1	40	166	26.4	(20.0, 34.0)	1		0.42		
	Quadrant 2	83	269	30.1	(25.7, 36.5)	1.2	(0.8, 1.9)			
	Quadrant 3	51	168	29.9	(23.4, 37.3)	1.2	(0.7, 1.9)			
	Quadrant 4	28	74	37.5	(26.9, 49.4)	1.7	(0.9, 3.1)			
Gender of HH head	Female	23	81	28.5	(19.6, 39.5)	1		0.72		
	Male	179	596	30.5	(26.9, 34.4)	1.1	(0.64,1.86)			
Age of the HH head	≤35	60	233	25.8	(20.5, 31.8)	1		0.147	1	
	36–40	41	127	32.3	(24.6, 41.1)	1.4	(0.8, 2.2)		0.8	(0.5, 1.5)
	41–50	60	169	36.4	(29.3, 44.0)	1.7	(1.1, 2.6) *		0.9	(0.5, 1.6)
	>51	41	148	28.6	(21.8, 36.7)	1.2	(0.7, 1.9)		0.9	(0.4, 1.7)
Education of HH	Above primary	15	94	16.7	(10.2, 26.1)	1		0.001	1	
	Primary	27	116	23.6	(16.7, 32.3)	1.5	(0.8, 3.1)		1.4	(0.6, 2.9)
	No education	160	467	34.7	(30.5, 39.1)	2.6	(1.5, 4.8) **		2.3	(1.2, 4.4) *
Occupation of HH head	Farmer	195	655	30.2	(26.8, 33.8)	1		0.56		
	Government employee	4	15	24.6	(9.3, 50.8)	0.8	(0.2, 2.4)			
	Other	3	7	47.4	(16.7, 80.1)	2.1	(0.5, 9.5)			
Marital status of HH head	Married	189	614	31.2	(27.6, 35.0)	1		0.28		
	Widowed	9	43	21.1	(11.1, 36.2)	0.6	(0.3, 1.3)			
	Single/divorced	4	20	21.2	(18.1, 35.1)	0.6	(0.2, 1.8)			
History of malaria in the past year of HH head	No	159	559	28.7	(25.0, 32.6)	1		0.06	1	
	Yes	43	118	37.7	(29.2, 46.9)	1.5	(0.9,2.3)		1.1	(0.6, 2.0)
Family size	1–3 persons	7	117	6.5	(3.1, 13.1)	1		<0.001	1	
	4–6 persons	120	388	31.4	(26.9, 36.1)	6.6	(2.9, 14.7) **		7.4	(2.6, 21.3) **
	≥7 persons	75	172	44	(36.6, 51.7)	11.3	(4.9, 26.0) **		2.5	(0.8, 8.2)
Predominant material in HH walls	Mud	200	669	30.3	(26.9, 33.9)	1		0.7		
	Cements	2/8	8	24	(5.7, 62.1)	0.7	(0.1, 3.8)			
Predominant material in HH roof	Iron	62	227	27.8	(22.2, 34.1)	1		0.32		
	Thatched	140	450	31.5	(27.4, 35.9)	1.2	(0.8, 1.7)			
Firewood use for cooking	No	102	382	27.4	(23.1, 32.1)	1		0.08	1	
	Yes	100	295	33.9	(28.6, 39.6)	1.4	(0.9, 1.9)		1.4	(0.9, 2.2)
Livestock ownership	No	7	33	22.4	(10.9, 40.6)	1		0.34		
	Yes	195	644	30.6	(27.2, 34.3)	1.5	(0.6, 3.7)			
History of malaria in the past year of any household member	No	165	583	28.5	(24.9, 32.3)	1		0.01	1	
	Yes	37	94	41.6	(31.9, 52.0)	1.8	(1.1, 2.8) *		1.4	(0.7, 2.6)
Discussion about malaria in the past month	No	47	137	34	(26.4, 42.5)	1		0.28		
	Yes	155	540	29.2	(25.6, 33.2)	0.8	(0.5, 1.2)			
HH access to LLINs	No	95	114	83.25	(75.12, 89.11)	20.3	(11.9, 34.9) **	<0.01	34.0	(17.1, 67.5) **
	Yes	107	563	19.63	(16.54, 23.14)	1			1	

* *p* < 0.05; ** *p* < 0.001.

## Supplementary Materials

The following are available online at www.mdpi.com/1660-4601/14/11/1312/s1, Table S1: Demographics, household characteristics and history of malaria by quadrants in the study area, southwest Ethiopia.
