# Supplementary material for: Long-Lasting Insecticide Net Ownership, Access and Use in Southwest Ethiopia: A Community-Based Cross-Sectional Study
Source: Int J Environ Res Public Health. 2017 Oct 27;14(11):1312. doi: 10.3390/ijerph14111312 (PMC5707951; doi:10.3390/ijerph14111312)
Supplement: Supplementary file 1 [file ijerph-14-01312-s001.pdf]

Table S1. Demographics, household characteristics and history of malaria by quadrants in the study area, southwest Ethiopia.

Covariates		Quadrants				
		Q1	Q2	Q3	Q4	Total
		n (%)	n (%)	n (%)	n (%)	n (%)
**Gender of HH head**	Female	22 (11.0)	36 (12.2)	31 (15.9)	14 (13.7)	103 (13.1)
	Male	182 (89.0)	270 (87.8)	173 (84.1)	88 (86.3)	713 (86.9)
**Age of the HH head (years)**	<= 35	62 (30.4)	107 (34.3)	70 (32.9)	29 (29.0)	268 (32.4)
	36-40	35 (17.3)	53 (17.3)	42 (20.5)	17 (16.5)	147 (18.1)
	41-50	54 (25.6)	76 (25.0)	47 (23.3)	28 (26.7)	205 (24.9)
	>51	53 (26.7)	70 (23.4)	45 (23.3)	28 (27.8)	196 (24.6)
**Education of HH**	Above primary	38 (16.5)	28 (10.2)	16 (8.5)	17 (17.9)	99 (12.1)
	Primary	50 (25.6)	25 (8.4)	55 (26.6)	12 (10.7)	142 (17.6)
	No education	116 (57.9)	253 (81.4)	133 (64.9)	73 (71.4)	575 (70.3)
**Occupation of HH head**	Farmer	191 (93.6)	299 (97.5)	200(98.0)	99 (96.9)	789 (96.6)
	Government employee	10 (4.7)	4 (1.5)	2 (0.8)	1 (0.8)	17 (2.0)
	Other	3 (1.7)	3 (1.0)	2 (1.2)	2 (2.3)	10 (1.4)
**Marital status of HH head**	Married	178 (86.8)	277 (90.0)	181 (88.2)	93 (91.4)	729 (89.0)
	Widowed	12 (6.3)	20 (6.7)	15 (7.8)	7 (6.7)	54 (6.9)
	Single/divorced	14 (6.9)	9 (3.3)	8 (4.0)	2 (1.9)	33 (4.1)
**History of malaria in the past year of HH head**	No	154 (75.9)	270 (87.7)	165 (81.0)	75 (75.2)	664 (81.7)
	Yes	50 (24.1)	36 (12.3)	39 (19.0)	27 (24.8)	152 (18.3)
**Family size**	1-3 persons	34 (16.8)	53 (17.3)	37 (17.9)	14 (13.7)	138 (17.0)
	4-6 persons	118 (59.3)	165 (53.9)	114 (55.2)	54 (52.9)	451 (55.4)
	>=7 persons	52 (23.9)	88 (28.8)	53 (26.9)	34 (33.4)	227 (27.6)
**Predominant material in HH walls**	Mud	200 (98.2)	306 (100)	199 (96.9)	100 (98.0)	805 (98.5)
	Cements	4 (1.8)	-	5 (3.1)	2 (2.0)	11 (1.5)
**Predominant material in HH roof**	Iron	101 (51.3)	94 (31.8)	52 (26.2)	34 (31.9)	281 (34.8)
	Thatched	103 (48.7)	212 (68.2)	152 (73.8)	68 (68.1)	535 (65.2)
**Firewood use for cooking**	No	123 (58.9)	151 (49.1)	102 (50.9)	60 (60.7)	436 (53.2)
	Yes	81 (41.1)	155 (50.9)	102 (49.1)	42 (39.3)	380 (46.8)
**Livestock ownership**	No	15 (7.9)	6 (1.9)	12 (6.3)	7 (7.0)	40 (5.1)
	Yes	189 (92.1)	300 (98.1)	192 (93.7)	95 (93.0)	776 (94.9)
**History of malaria in the past year of any household member**	No	164 (82.1)	277 (90.3)	165 (81.4)	87 (85.9)	693 (85.4)
	Yes	40 (17.9)	29 (9.7)	39 (18.6)	15 (14.1)	123 (14.6)
**Discussion about malaria in the past month**	No	40 (20.8)	69 (22.3)	50 (26.1)	24 (25.8)	183 (23.4)
	Yes	164 (79.2)	237 (77.7)	154 (73.9)	78 (74.2)	633 (76.6)

Table S2. The surveyed population and LLIN ownership, access and use indicators in study villages, Southwest Ethiopia.

Quadrants	Village	People surveyed (n)	I1	I2	I3	I4	I5	I6
**Quadrant 1**	1	306	56.9	41.2	46.7	72.4	38.2	93.8
	2	270	90.2	78.4	82.4	86.9	74.4	97.3
	3	231	86.3	74.5	80.9	86.4	67.1	80.0
	4	263	92.2	82.4	86.7	89.4	76.0	90.9
**Quadrant 2**	5	257	96.1	88.2	91.5	91.8	85.2	94.0

	6	264	82.4	64.7	73.4	78.6	63.6	93.4
	7	273	74.5	60.8	67.4	81.6	57.5	87.8
	8	268	86.3	64.7	75.2	75.0	63.8	93.8
	9	267	96.1	80.4	89.9	83.7	78.2	85.5
	10	285	92.2	66.7	79.4	72.3	63.2	85.4
**Quadrant 3**	11	258	88.2	78.4	82.2	88.9	74.8	90.9
	12	288	90.2	70.6	80.9	78.3	72.6	94.0
	13	277	64.7	54.9	58.8	84.8	44.0	91.6
	14	235	86.3	76.5	81.2	88.6	70.2	87.6
**Quadrant 4**	15	286	70.5	64.7	66.5	91.7	53.5	80.6
	16	295	74.5	56.9	61.9	76.3	47.1	87.5

I: Calculated indicators of LLIN Coverage, Access and Use; n: number of Ind.
